# Constructive *Q*-Matrix Identifiability via Novel Tensor Unfolding

**DOI:** 10.1017/psy.2025.10078

**Published:** 2026-01-06

**Authors:** Yuqi Gu

**Affiliations:** Department of Statistics, Columbia University, USA

**Keywords:** Algebraic statistics, Cognitive diagnostic model, Constructive proof, Identifiability, Q-matrix, Tensor unfolding

## Abstract

This work establishes a new identifiability theory for a cornerstone of various cognitive diagnostic models (CDMs) popular in psychometrics: the Q-matrix. The key idea is a novel tensor-unfolding proof strategy. Representing the joint distribution of *J* categorical responses as a *J*-way tensor, we strategically unfold the tensor into matrices in multiple ways and use their rank properties to identify the unknown Q-matrix. This approach departs fundamentally from all prior identifiability analyses in CDMs. Our proof is constructive, elucidating a population-level procedure to exactly recover the Q-matrix within a parameter space where each latent attribute is measured by at least two “pure” items that solely measure this attribute. The theory has several desirable features: it can constructively identify both the Q-matrix and the number of latent attributes; it applies to broad classes of linear and nonlinear CDMs with main or all saturated effects of attributes; and it accommodates polytomous responses, extending beyond classical binary response settings. The new identifiability result unifies and strengthens identifiability guarantees across diverse CDMs. It provides rigorous theoretical foundations and indicates a future pathway toward using tensor unfolding for practical Q-matrix estimation.

## 1 Introduction

Cognitive Diagnostic Models (CDMs), or Diagnostic Classification Models (Rupp and Templin, 2008; von Davier and Lee, 2019), are popular discrete latent variable models widely used in educational and psychological measurement. Based on test respondents’ multivariate categorical item responses, one can use a CDM to infer their fine-grained discrete latent attributes, such as cognitive abilities, personality traits, or mental disorders. One key structure in a CDM is the *Q*-matrix (Tatsuoka, 1983), which is a 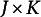 binary matrix describing how the *J* items depend on the *K* latent attributes. Various CDMs have been proposed with different modeling assumptions. Popular examples include the conjunctive Deterministic Input Noisy Output “And” gate model (DINA; Junker and Sijtsma, 2001), the disjunctive Deterministic Input Noisy Output “Or” gate model (DINO; Templin and Henson, 2006), the main-effect diagnostic models including the linear logistic models (LLM; Maris, 1999), the reduced Reparameterized Unified Model (reduced-RUM; DiBello et al., 2012), and the additive CDM (ACDM; de la Torre, 2011), and all-saturated-effect diagnostic models including the general diagnostic models (GDM; von Davier, 2008), the log-linear CDM (LCDM; Henson et al., 2009), and the generalized DINA model (GDINA; de la Torre, 2011).

The identifiability of CDMs is crucial to ensuring the validity of their statistical analysis. Especially in exploratory CDMs where the *Q*-matrix is unknown, uniquely identifying *Q* is a fundamental prerequisite for reliably estimating it using any method. In the past decade, the identifiability problems of CDMs and *Q*-matrix have attracted increasing interest. Regarding the *proof techniques*, the existing identifiability studies fall into two categories. The first category (Chen et al., 2020; Culpepper, 2019,2; Fang et al., 2019; Liu and Culpepper, 2024) leveraged Kruskal’s Theorem on the uniqueness of three-way tensor factorizations (Kruskal, 1976,7) to establish identifiability. These approaches share a similar spirit to the general proof framework popularized by Allman et al. (2009). The second category (Chen et al., 2015; Gu and Xu, 2021; Liu et al., 2013; Xu and Shang, 2018) exploited the marginal moments of the binary responses and investigated the uniqueness of roots to the polynomial equations defined by these moments. This fine-grained proof technique can sometimes deliver sharper identifiability conditions compared to directly invoking Kruskal’s Theorem.

In contrast to all of the above existing work, we adopt a fundamentally different proof strategy based on *tensor unfolding*. Unfolding or flattening a tensor (third-order or higher-order array) means reshaping it into a lower-order tensor or a matrix (Kolda and Bader, 2009). In a CDM, the population distribution of the *J* observed categorical variables can be represented by a *J*th-order tensor, whose entries are elements in the joint probability mass function of the *J*-dimensional response vector. Our key proof idea is to strategically unfold this tensor to a matrix in various ways and use the rank properties of these resulting matrices to uniquely identify the *Q*-matrix. Our identifiability proof is a *constructive proof* for the first time in the literature: the proof itself is a population-level procedure and algorithm to reconstruct the *Q*-matrix from the population distribution in a restricted identifiable *Q*-matrix space. This identifiable space contains all *Q*-matrices that have at least two “pure” items solely measuring each latent attribute. Our proof’s constructive nature departs from all existing identifiability analyses of the *Q*-matrix: previous works adopt *existence proofs*, by proving that if two *Q*-matrices lead to the same population distribution of observed responses, the two *Q*-matrices must be identical, without indicating how the *Q*-matrix can be reconstructed from the population distribution.

Our identifiability result enjoys three additional features. First, it can directly and constructively *identify the number of latent attributes K* together with the *Q*-matrix. Second, it is broadly applicable to various flexible CDMs, including all main-effect CDMs (such as reduced-RUM, LLM, and ACDM) and all-saturated-effect CDMs (such as GDM, LCDM, and GDINA). Third, it applies to CDMs with polytomous responses, in addition to traditional CDMs with binary responses. Recently, variants of CDMs with polytomous responses (Culpepper, 2019; Fang et al., 2019; Liu and Culpepper, 2024; Ma and de la Torre, 2016; Wayman et al., 2025) have attracted increasing interest due to their flexibility. Our theory offers a unified identifiability result for all these CDMs with general categorical responses. Compared to existing identifiability conditions for binary- or polytomous-response CDMs, our new condition is weaker because it only requires the *Q*-matrix to contain two identity matrices 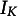 without any additional assumption. Specifically, this implies that if there are exactly 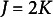 items and the *Q*-matrix contains just two 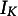 , then this *Q* is still identifiable; whereas to our best knowledge, such a *Q*-matrix does not satisfy previous identifiability conditions for general CDMs.

In the rest of this article, Section 2 introduces the unfoldings of the probability tensor in CDMs and gives a detailed illustrative example. Section 3 presents the new identifiability theory for both binary CDMs and polytomous CDMs. Section 4 concludes and discusses a future direction. The proofs of the theoretical results are included in the Appendix.

## 2 Unfolding Population Distribution Tensors in a CDM

### 2.1 Model Setup of CDMs with Binary Responses and Attributes

Consider a CDM where each subject is associated with *J* observed variables 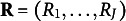 as responses to *J* test items and *K* latent attributes 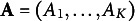 . We start by considering the most commonly used CDMs with binary responses 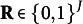 and binary attributes 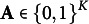 , and later will generalize our result to polytomous responses and attributes. A key structure in a CDM is the *Q*-matrix introduced in Tatsuoka (1983), which specifies the relationship between the observed responses and the latent attributes. The *Q*-matrix is a 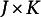 matrix 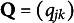 with binary entries, with rows indexed by the *J* items and columns by the *K* attributes. The entry 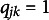 or 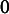 indicates whether or not the *j*th item requires/measures the *k*th latent attribute. The row vectors of the *Q*-matrix are also called the 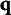 -vectors and denoted by 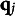 for each 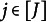 . Here, for any positive integer *M*, we denote 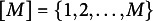 .

To model the conditional distribution of the observed 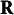 given the latent 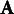 , we use the general notation of the *item parameters*


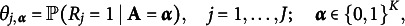

and denote the collection of them by 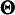 . These item parameters are subject to certain equality constraints imposed by the *Q*-matrix in different ways under different models; see Examples 1 and 2 for concrete examples. For the latent attributes, we consider the most commonly adopted saturated attribute model with proportion parameters 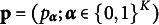 , where 

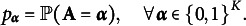

The proportion parameters 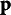 satisfy that 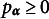 for all 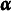 and 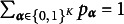 . We use 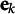 to denote the *K*-dimensional canonical basis vector with an “1” in the *k*th entry and “0” in all other entries.

Under the above general setup, various CDMs were proposed with different diagnostic modeling assumptions and different link functions. *In this work, we focus on the flexible main-effect or all-saturated-effect CDMs*, which are also called the multi-parameter restricted latent class models in Gu and Xu (2021) and encompass a wide range of diagnostic models.

Remark 1. The Boolean-product-based DINA and DINO models (also called two-parameter restricted latent class models) exhibit very different algebraic structures from the multi-parameter CDMs. Such distinctive properties make the marginal-moment-based proof technique (Gu and Xu, 2021; Xu and Zhang, 2016) optimal for deriving the minimal identifiability conditions for the DINA and DINO models. Necessary and sufficient identifiability conditions for the *Q*-matrix in these models are already well understood in the literature (Gu, 2023; Gu and Xu, 2021). Therefore, we do not consider the DINA and DINO models in this work and instead focus on the general and flexible main-effect or all-saturated-effect CDMs.

In the following, we review concrete examples of multi-parameter CDMs in the following.

Example 1 **(Main-Effect Cognitive Diagnosis Models).**

An important family of cognitive diagnosis models assumes that the 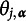 depends on the main effects of those attributes required by item *j*, but not their interactions. This family includes the popular reduced Reparameterized Unified Model (rRUM; DiBello et al., 2012), Additive Cognitive Diagnosis Models (ACDM; de la Torre, 2011), the Linear Logistic Model (LLM; Maris, 1999), and the General Diagnostic Model (GDM; von Davier, 2008). We call them the Main-Effect Cognitive Diagnosis Models. In particular, under the rRUM, 

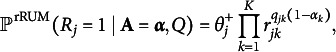

where 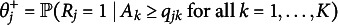 represents the positive response probability of a capable subject of item *j* (i.e., a subject mastering all required skills of item *j*), and 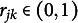 is the parameter penalizing not mastering attribute *k* required by item *j*. Equivalently, the item parameter in the rRUM can be written as 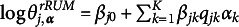 . Similarly, the ACDM assumes the parameter 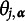 can be written as a linear combination of the main effects of the required attributes: 

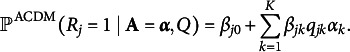

The Linear Logistic Model assumes a logistic link function with 

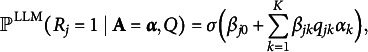

where 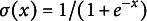 is the logistic function.

Example 2 **(All-saturated-effect Cognitive Diagnosis Models).**

Another popular type of cognitive diagnostic model assumes that the positive response probability depends on the main effects and all of the interaction effects of the required attributes of the item. We call these models all-saturated-effect cognitive diagnosis models. The GDINA model (de la Torre, 2011), the log-linear cognitive diagnosis models (LCDM; Henson et al., 2009), and the general diagnostic model (GDM; von Davier, 2008) are popular examples of all-saturated-effect models. The item parameters (conditional positive response probabilities) under these models are (1)

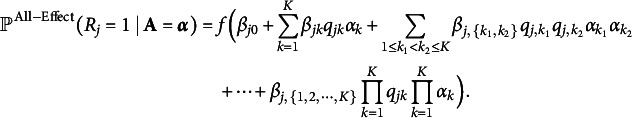

When *f* is the identity link 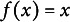 , the above gives the GDINA model, and when *f* is the inverse logit link 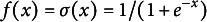 , the above gives the LCDM. von Davier (2014) showed that the GDINA and LCDM can be rewritten as GDMs with an extended skill space.

The local independence assumption is usually imposed in CDMs, meaning that the observed responses 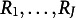 are conditionally independent given the latent attributes 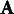 . With this assumption, we can write the joint distribution of the observed 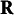 by marginalizing out the latent 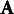 in a CDM with binary responses and binary attributes: (2)

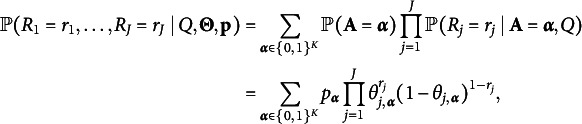

for any response pattern 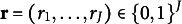 , where the *Q*-matrix-induced constraints on parameters 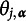 are made implicit. The *Q*-matrix is said to be identifiable, if it can be uniquely identified from the population distribution of 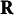 in (2) up to the column permutation of *Q* (Liu et al., 2013).

### 2.2 Population Distribution Tensor under a CDM

Studying the identifiability in CDMs often requires one to take the tensor perspective. This subsection introduces the population tensor under a CDM and explains its importance in studying model identifiability.

We first introduce the notation of the *population distribution tensor* summarizing the population distribution of the observed response vector 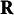 . The distribution of 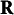 in (2) can be characterized by a *J*th-order tensor 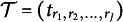 of size 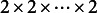 with entries (3)

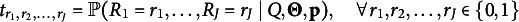

denotes the marginal probability of the response pattern 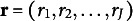 under the specified CDM. Tensor 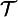 is said to have *J modes* indexed by the items 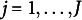 . We call 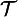 the *population distribution tensor* of 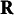 . For a vector 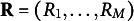 and a subset 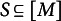 , denote 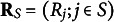 as a 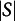 -dimensional subvector of 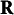 that collects all entries indexed by *S*.

When establishing the identifiability of the *Q*-matrix or continuous parameters in main-effect or all-saturated-effect CDMs, previous proof approaches based on Kruskal’s Theorem often involve a step of rewriting this *J*th-order tensor 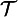 into a three-way tensor (third-order tensor) by concatenating certain modes together (see, e.g. Culpepper, 2019; Fang et al., 2019). Even for studies that do not explicitly invoke Kruskal’s Theorem (see, e.g. Gu and Xu, 2020; Xu, 2017; Xu and Shang, 2018), the identifiability proofs still implicitly partition the *J* binary responses into three subsets and investigate the polynomial moment equations arising from this partitioning. In this work, we take a fundamentally different approach but still investigates the population tensor 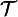 . Our approach *avoids looking at three-way* tensor decompositions but examines *two-way tensor unfoldings* and hence delivers a strictly more relaxed identifiability condition for multiparameter CDMs than existing studies; the next subsection presents details of the tensor unfolding.

### 2.3 Tensor Unfoldings

This subsection introduces the key *tensor unfolding* idea behind our new identifiability proof. Unfolding or flattening a tensor (third-order or higher-order array) means reshaping it into a lower-order tensor or a matrix (Kolda and Bader, 2009). To prove the identifiability result for the *Q*-matrix, we show that it suffices to consider unfolding the *J*th-order tensor 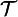 to various matrices. For this purpose, define the *row group* of indices as 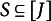 and the *column group* as 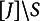 . We denote the matrix resulting from the unfolded tensor as (4)

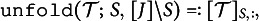

which is a 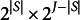 matrix with rows indexed by the 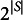 configurations of 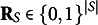 and columns by the 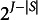 configurations of 
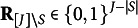
 . The entries in 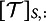 are still those marginal response probabilities 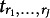 in (3). A rather surprising fact that we will uncover shortly is that the ranks of such matrices (4) contain rich information about the unknown *Q*-matrix. For example, when *S* contains two items, the rank of 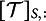 reflects whether these two items solely measure the same latent attribute; see Section 2.4 for details. So, we will strategically unfold the tensor 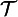 and use the rank properties of the resulting matrices as certificates to identify and recover the unknown *Q*-matrix.

We also introduce the notion of *marginal tensor* and *marginal unfolding*. Given a subset 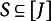 , define the *marginal probability tensor* for 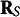 as a 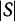 -way tensor 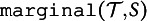 with entries specifying the joint PMF of the random vector 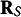 . This marginal tensor can be obtained from the aforementioned full tensor 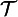 by appropriately summing up its entries: each new entry in 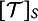 takes the form of 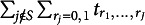 . So, 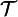 and *S* uniquely defines the marginal probability tensor for 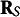 and we denote it by 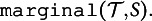 For two disjoint index sets 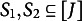 whose union does not equal 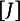 , we define a *marginal unfolding*


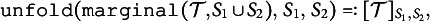

which characterizes the joint probability table between random vectors 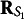 and 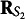 . The entries in 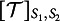 are summations of 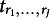 in the form of 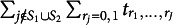 , corresponding to the PMF of 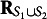 by marginalizing out other random variables 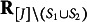 . We introduce the marginal tensor 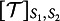 in order to focus on the items belonging to 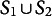 and exclude the effects of latent attributes not manifested in 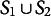 .

We introduce the definition of a *conditional probability table* (CPT). Let 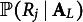 denote the CPT that specifies the conditional distribution of 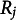 given the latent attributes 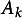 for 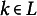 . In the general case where each observed 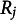 has *C* categories, the CPT 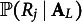 is a matrix with size 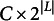 . Its 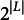 columns are indexed by all possible configurations of the latent vector 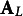 ranging in 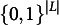 , and its *C* rows are indexed by the *C* categories of 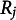 in 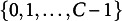 .

Finally, we introduce a useful notation of the *joint probability table* between two random vectors. Consider general categorical latent 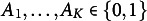 . For two sets 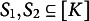 , we describe the joint distribution of 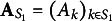 and 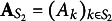 using 

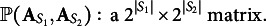

The rows of 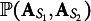 are indexed by the configurations of 
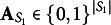
 , and columns by those of 
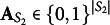
 . Importantly, we emphasize that 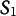 and 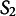 *do not need to be disjoint and can overlap*. At a high level, allowing overlapping sets will enable us to study the rank properties of unfoldings potentially involving multi-attribute items. When 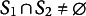 , 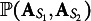 has some zero entries corresponding to impossible configurations of 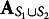 . An extreme example is if 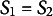 , then 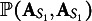 is a 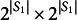 diagonal matrix, because 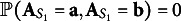 for any 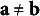 . In this case, the diagonal entries of 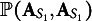 are given by the PMF of 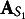 . Another example is when 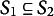 , then the matrix 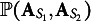 has orthogonal row vectors because 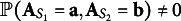 only if 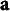 is a subvector of 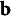 indexed by integers in 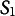 . This general matrix notation of 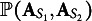 for potentially overlapping sets 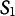 and 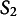 turns out to be very useful to facilitate our identifiability proofs.

### 2.4 A Toy Example Illustrating the Tensor Unfolding Insight

The next toy example illustrates the tensor unfolding idea and reveals useful insights on how to utilize unfoldings to identify and recover the *Q*-matrix.

Consider 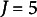 , 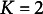 , and the following *Q*-matrix 

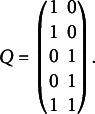

If considering binary responses 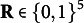 , then the population distribution tensor 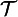 has size 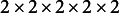 . Following the definition of CDMs in Section 2.1, we can write (5)

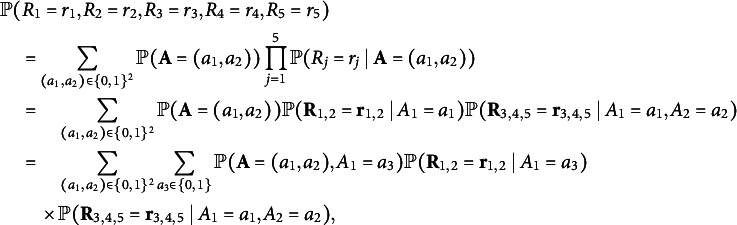

where we have purposefully introduced the term 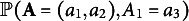 to facilitate the following matrix factorization perspective for the unfolded tensor. To be more specific, the last equality in the above expression holds because although 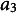 can freely range in 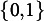 , the probability 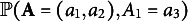 will be zero for any 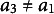 .

First, we unfold the tensor 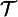 so that the row group contains item indices 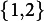 , and the column group contains item indices 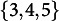 . Let 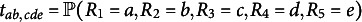 denote entries from the joint distribution of the five observed variables, where 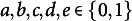 . With the row group 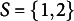 and column group 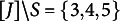 , the unfolded tensor is a 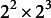 joint probability table of 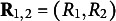 and 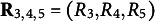 : (6)

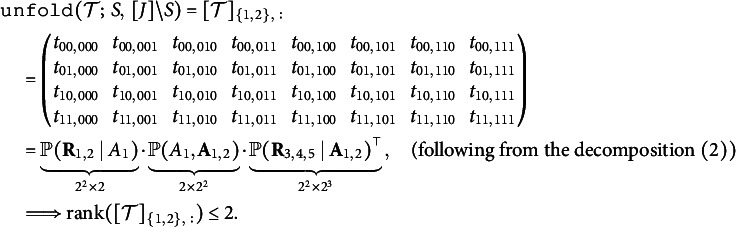

Specifically, 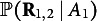 is a 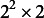 matrix that collects the following conditional probabilities: 

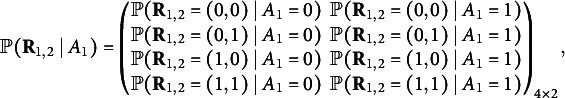

and the 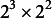 matrix 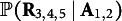 is defined similarly. In summary, the above derivation shows that we can factorize 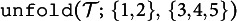 into the product of three matrices, which have size 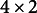 , 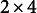 , and 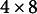 , respectively. Therefore, the rank of the 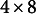 matrix 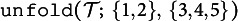 is at most 2. Similarly, 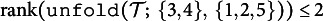 also holds due to symmetry because items 3 and 4 both solely measure the second attribute.

In contrast, if two items 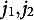 do not solely measure the same latent attribute, the unfolding 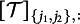 will show distinct rank behavior. Specifically, if we unfold the tensor 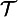 with the row group being 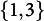 and the column group being 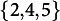 , then as will be shown rigorously in the proof of the main theorem, the unfolding 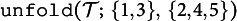 has rank strictly larger than 2; more generally, 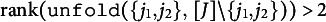 holds as long as items 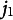 and 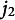 are not solely measuring the same attribute. In other words, whether the rank of 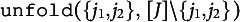 exceeds two is a perfect certificate that reveals whether 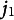 and 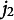 are measuring the same latent attribute. By exhaustively examining the rank of the unfoldings with the row group consisting of every pair of items, we can exactly tell which items are solely measuring the same attribute as well as the number of latent attributes: items 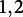 solely measure one latent attribute, items 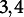 solely measure the other latent attribute, and there are two latent attributes. Note that if there is an additional item 6 that solely measures the first latent attribute, then 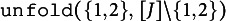 , 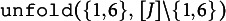 , and 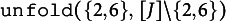 all have ranks at most two, so all three items 1, 2, and 6 will be correctly assigned as solely measuring the same latent attribute.

Since item 5 with 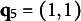 measures both latent attributes, identifying such 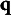 -vectors calls for a more challenging analysis than the pairwise unfolding illustrated in the previous paragraph. But surprisingly and fortunately, we rigorously prove (in Theorems 1 and 2 in Section 3) that examining strategically constructed unfolded matrices’ ranks can still identify the 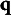 -vectors that vary arbitrarily in 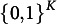 . The proof for these multi-attribute items is much more technical than the single-attribute items described above, so we omit the details here and refer interested readers to the proof of the theorems.

In summary, the toy example above illustrates that the ranks of unfolded tensors can help reveal the underlying *Q*-matrix. Our theorem applies to general *Q*-matrix dimensions and structures far beyond this toy example, as will be laid out in the next section.

## 3 Identifiability Theory

### 3.1 CDMs with Binary Responses

We next rigorously formalize the mild technical assumptions we impose for identifiability. If an item *j* solely measures attribute *k* (that is, 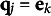 where 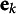 is the *k*th canonical basis vector), then we call it a *single-attribute item*.

Definition 1 **(Non-degenerate Single-attribute Items: Binary-CDMs).**

A single-attribute item *j* measuring attribute *k* is non-degenerate if the CPT 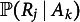 has full rank 2.

Requiring an item to satisfy Definition 1 is a mild assumption. Mathematically, if item *j* solely requires attribute *k*, then 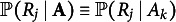 and the 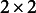 conditional probability table is: 

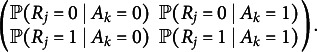

In this case, requiring 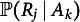 to have full rank 2 as specified in Definition 1 is equivalent to requiring 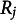 not to be independent of 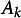 . We next present the first main theorem.

Theorem 1. Consider any CDM in Examples 1 and 2 with binary responses. Consider the following identifiable parameter space for the *Q*-matrix: (7)

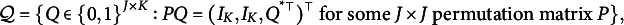

where 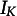 is the 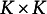 identity matrix and 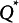 is an arbitrary binary matrix or an empty matrix (meaning 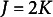 ). Assume all single-attribute items are non-degenerate and 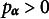 for all 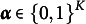 . If the true and alternative *Q*-matrices 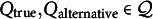 lead to the same marginal distribution of the observed response vector 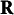 , then 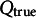 is identifiable and 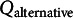 must be identical to 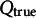 up to a column permutation. Moreover, 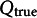 can be constructively identified from the population distribution tensor 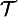 via strategically unfolding it and examining the ranks.

The identifiable *Q*-matrix space in (7) includes all 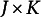 binary matrices that contain at least two identity submatrices after some column and row permutation. Specifically, we prove Theorem 1 by establishing the following two key technical propositions, which explicitly lay out the tensor unfolding procedures along with the corresponding rank certificates to identify and recover the *Q*-matrix.

Proposition 1. Under the condition in Theorem 1, for arbitrary 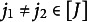 , it holds that 

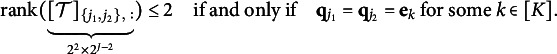

Proposition 1 formalizes the intuition explained in the toy example in Section 2.4, and precisely characterizes how to identify the 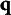 -vectors for all single-attribute items in the *Q*-matrix. In words, Proposition 1 states that the rank of 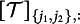 reflects whether the two items 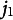 and 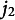 solely measure the same latent attribute. Interestingly, Proposition 1 also implies we can constructively identify *K*, the number of latent attributes: *K* is equal to the maximum number of mutually disjoint item sets 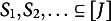 such that the rank of the unfoldings 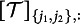 is at most two when considering all 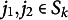 for each *k*.

Remark 2. In principle, the tensor unfolding techniques can also be applied to the DINA and DINO models, but the Boolean product structure in these two models (i.e., the conjunctive assumption in DINA and the disjunctive assumption in DINO) makes the rank properties of the unfolded tensor different from those under the main-effect and all-saturated-effect CDMs. More specifically, take Proposition 1 for example, which states that for arbitrary 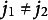 it holds that 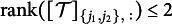 if and only if 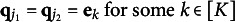 . Actually, under the DINA and DINO models, the “if” part in the above statement still holds (that is, the single-attribute items still lead to low-rank unfoldings), but the “only if” part no longer holds because of the Boolean-product structure (that is, the low-rank unfoldings are not necessarily caused by those single-attribute items). Therefore, under the DINA or DINO model, the rank of 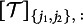 being at most 2 is not a valid “certificate” for concluding that items 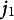 and 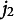 both only measure the same latent attribute. Based on the above reason, we focus on the non-DINA/DINO CDMs, for which the tensor unfolding technique can weaken the existing identifiability conditions of the Q-matrix and provide new theoretical understanding.

Having identified *K* and all single-attribute items, the next proposition characterizes a more nuanced tensor unfolding strategy along with their rank certificates to identify the challenging multi-attribute items. These items can have arbitrary corresponding 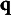 -vectors ranging in 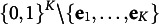 .

Proposition 2. Under the condition in Theorem 1, assume without loss of generality that the first 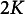 rows of the *Q*-matrix are identified to be 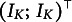 . The following holds for any attribute 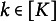 and any item *j* that requires at least two attributes (i.e., with 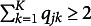 ): 

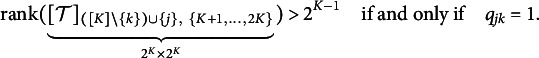

The proof of Proposition 2 is challenging. The difficulties include: first, to come up with an appropriate row group 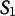 and a corresponding column group 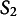 of variables from 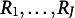 such that the marginal unfolding 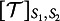 contains as much as possible useful information about the multi-attribute 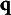 -vectors; and second, to carefully investigate the rank of these unfoldings to establish the certificate for recovering those 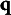 -vectors.

The identifiability condition in Theorem 1 is weaker than existing strict identifiability conditions on the *Q*-matrix for main-effect or all-saturated-effect, binary-response or polytomous response CDMs (e.g., Culpepper, 2019; Fang et al., 2019; Xu and Shang, 2018). Specifically, the condition in Theorem 1 is satisfied if, after some row permutation, *Q* takes the form of 
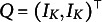
 with exactly 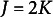 items; that is, when each latent attribute is exactly measured by two pure items, and there are no additional items in the test. To our best knowledge, such a *Q*-matrix does not satisfy the identifiability condition in the existing studies for general CDMs. The high-level intuition behind why our condition relaxes existing conditions in the literature is that, existing studies either explicitly or implicitly leverage the *three-way* decompositions of the population distribution tensor to establish identifiability. In contrast, we work with the rank of *matrices* to establish identifiability despite starting with tensors, and these matrices essentially describe the joint distributions of *two* random vectors instead of *three* random vectors. This intuitively explains why our approach delivers a weaker identifiability condition that only requires each latent attribute to be measured twice in the *Q*-matrix.

It is worth noting that Culpepper (2023) relaxed the condition that *Q* needs to contain identity submatrices by introducing and studying the notion of “dyads”, which consist of pairs of items. Specifically, the identifiability theorem in Culpepper (2023) applies even if no single-attribute items exist by allowing *saturated dyads*. This significantly broadens the applicability of the identifiability theory to practical cognitive diagnostic assessment settings. However, that work still leverages Kruskal’s Theorem on the uniqueness of three-way tensor decompositions to establish identifiability with the dyad structure and requires 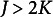 . So, the theorem in Culpepper (2023) does not apply to the case where the *Q*-matrix contains exactly 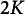 rows; moreover, the proof of that theorem is still an existence proof, instead of a constructive proof like our tensor unfolding approach.

### 3.2 CDMs with Polytomous Responses

In this section, we consider CDMs with polytomous item responses. Suppose item 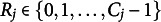 has 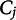 categories for each 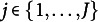 , where 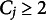 is a general integer. Here, we allow different items to have different numbers of response categories (i.e., 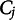 across different *j* can be different), similar to the setting considered in Liu and Culpepper (2024) and Wayman et al. (2025). We still use the same notation for the proportion parameters 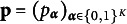 to describe the joint distribution of the binary latent attributes. To describe the conditional distribution of the responses 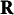 given the latent attributes 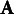 , we introduce item parameters 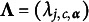 : 

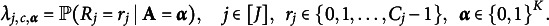

The *Q*-matrix induces constraints on the 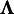 parameters similar to the binary CDM case; here, 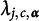 depends only on those attributes that are measured by the *j*th item according to the *Q*-matrix. Mathematically, this means 

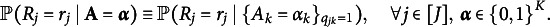

The above general assumption summarizes the key property shared by many polytomous-response CDMs. Our considered setting hence covers existing models for polytomous responses, such as Chen and de la Torre (2018), Fang et al. (2019), Culpepper (2019), and Liu and Culpepper (2024).

The population distribution of the observed response vector 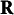 can be written as 

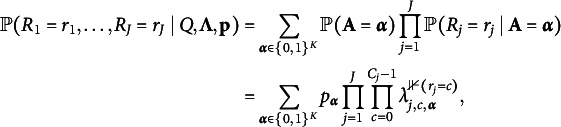

for any multivariate polytomous response pattern 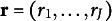 with 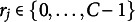 for each *j*. The *J*-way population distribution tensor 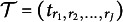 characterizing the distribution of 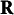 has size 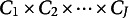 , with entries 

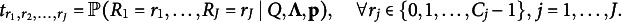

We will unfold this tensor similarly to the binary CDM case. Consider the *row group* of indices as 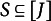 and the *column group* as 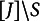 , then the matrix resulting from the unfolded tensor is 

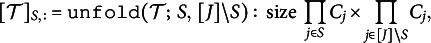

The notations of marginal tensors and marginal unfoldings are defined similarly.

To formalize the requirements on the polytomous CDMs, we introduce the following two definitions.

Definition 2 **(Non-degenerate Single-attribute Items in Polytomous-CDMs).**

A single-attribute item *j* solely measuring attribute *k* is said to be non-degenerate if the following conditional probability table, denoted by 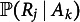 , has full column rank 2: 

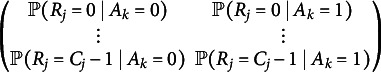

Definition 2 is a direct extension of the earlier Definition 1 to the polytomous CDMs and covers that as a special case.

As mentioned earlier in Section 2.1, we do not consider the Boolean-product-based CDMs (e.g., DINA or DINO models) in this work. We next formalize the non-DINA/DINO assumption in the polytomous CDM.

Definition 3 **(Non-DINA/DINO Assumption).**

For any response category 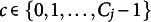 and any latent attributes configuration 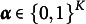 , the conditional probability 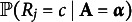 does not conform to the Boolean-product-based DINA or DINO model, but rather conforms to any linear or nonlinear main-effect or all-saturated-effect-based CDM.

We have the following main theorem for polytomous CDMs with essentially the same identifiability condition as the binary CDMs, again proved via tensor unfoldings.

Theorem 2 **(Polytomous-response CDMs).**

Consider a polytomous-response CDM under Definitions 2 and 3. Still consider the restricted identifiable *Q*-matrix space 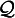 defined in (7). Assume all single-attribute items are non-degenerate and 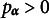 for all 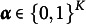 . If the true and alternative Q-matrices 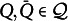 lead to the same distribution of the observed polytomous response vector 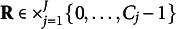 , then the *Q*-matrix is identifiable and can be constructively identified from the population distribution tensor 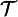 via unfoldings.

Theorem 2 is proved by establishing the following proposition on the ranks of the unfolded tensors in polytomous CDMs.

Proposition 3. Under the condition in Theorem 1, the following rank statements hold.

- For arbitrary 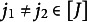 , it holds that 

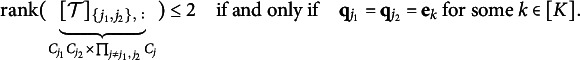

- Assume without loss of generality that the first 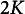 rows of the *Q*-matrix are identified to be 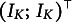 . The following holds for any attribute 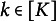 and any item 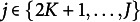 that requires at least two attributes (i.e., with 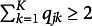 ): 

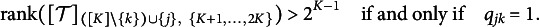

The above proposition generalizes Proposition 1 from the binary setting to the general polytomous setting. The two scenarios (a) and (b) in Proposition 3 give explicit and precise characterizations of the 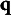 -vectors of single-attribute items and multiple-attribute items, respectively. These conditions are simple rank constraints of the matrices resulting from unfolding the 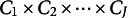 tensor 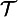 , and they serve as certificates to reveal the unknown 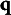 -vectors for all *J* items.

It is worth mentioning that prior works have studied *generic identifiability* in CDMs, which is a slightly weaker notion than strict identifiability studied in this work. Generic identifiability means the parameters are almost everywhere identifiable in the parameter space, possibly excluding a Lebesgue mesasure zero set where identifiability breaks down. For main-effect or all-saturated-effect CDMs, generic identifiablity has been established under weaker conditions on the *Q*-matrix (e.g., Chen et al., 2020; Gu and Xu, 2020), without requiring *Q* to contain two identity submatrices 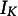 . We remark that it is not technically straightforward to establish generic identifiability using the tensor unfolding technique. To clarify, most previous studies that established generic identifiability for CDMs mainly utilized Kruskal’s Theorem by adapting the technique popularized by Allman et al. (2009). Roughly speaking, in those works, generic identifiability is often proved by finding one possible configuration of the parameters and showing that identifiability holds under this configuration using Kruskal’s Theorem; then, generic identifiability follows by concluding that the identifiable parameter configuration is “generic” in the parameter space, so that non-identifiability occurs only in a negligible subset of the parameter space. Therefore, generic identifiability is inherently tied to the Kruskal Theorem-based *existence proofs*, while not entirely aligned with the tensor-unfolding-based *constructive proofs*. Relaxing the condition of “*Q* contains at least two 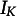 matrices” would distort the rank properties of the unfolded tensor, which are important certificates to recover the *Q*-matrix in our proof.

## 4 Discussion

We have established a new identifiability theory of the *Q*-matrix for various main-effect and all-saturated-effect, binary and polytomous-response CDMs. Our proof exploits a novel tensor unfolding technique and is the first constructive identifiability proof in the literature.

Previous studies such as Chen et al. (2020) and Liu and Culpepper (2024) leveraged Kruskal’s Theorem to study the “ 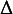 -matrix” in diagnostic models, which has size 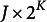 , with columns corresponding to both main-effects and all possible higher-order interaction effects of latent attributes. Compared to the *Q*-matrix, the 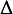 -matrix provides a more fine-grained summary of the measurement model structure and distinguishes the main-effect models (such as the ACDM and reduced-RUM) from the all-saturated-effect models (such as the GDINA and LCDM). On the other hand, however, the *Q*-matrix can be viewed as a more general and high-level summary of the *statistical dependence* relationship between the observed item responses and the latent attributes. In the language of probabilistic graphical models, the *Q*-matrix encodes the bipartite graph structure between the observed variables and the latent ones, where 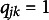 or 0 indicates whether or not the *j*th observed response *directly depends on* (has a directed arrow from) the *k*th latent attribute. One interesting property of our tensor-unfolding-based identifiability result is that the proof is *agnostic* to the measurement model structure, regardless of whether the model is main-effect or all-saturated-effect, and always recovers the fundamental dependence relationship between the observed and latent variables encoded by the *Q*-matrix. More specifically, the rank properties of the unfolded tensor reflect whether each observed variable depends on each latent variable, rather than the detailed form of this dependence (i.e., being main-effect only or involving interaction effects). With that said, we believe that it may be possible to adapt the tensor unfolding technique to study the identifiability of the 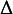 -matrix in the future.

To facilitate understanding the constructive proof, we next provide a summary of how we leverage tensor unfolding to uniquely identify and recover the *Q*-matrix entries based on the probabilities of the response patterns. This proof outline follows from Propositions 1 and 2 for binary-response CDMs, and Proposition 3 for polytomous-response CDMs. First, we identify all single-attribute items by exhaustively considering all pairs of items 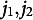 ranging in the item set 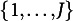 and examining the rank of the unfolding 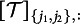 . If the rank is smaller than or equal to 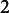 , then items 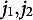 must be measuring the same latent attribute. After this first step, all 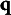 -vectors corresponding to single-attribute items have been recovered, giving *K* item sets 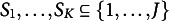 , where each set 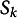 contains items that solely measure latent attribute *k*. Our identifiability condition ensures that each 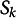 contains at least two items, and we denote these two item indices by 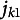 and 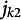 . Second, for any other item *j* that does not belong to 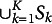 and for any 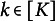 , we recover the entry 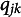 by considering the unfolding 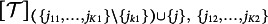 . If the rank of this unfolding is larger than 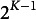 , then we conclude 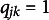 ; otherwise, conclude 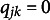 . After this second step, all 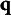 -vectors corresponding to multi-attribute items have also been recovered. This completes the constructive proof strategy for identifying and recovering the whole *Q*-matrix.

Our proof indicates an interesting future direction toward practically estimating the *Q*-matrix via tensor unfolding. To clarify, the identifiability proof in this paper operates on the *population distribution tensor* summarizing the population distribution of 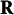 ; this identifiability notion is the same as the classical notion of population identifiability in all existing studies of CDMs and the *Q*-matrix (e.g., Chen et al., 2020,1; Culpepper, 2019; Gu and Xu, 2021; Liu et al., 2013; Xu and Shang, 2018). In practice, the available data are a finite sample, and one needs to use the samples to estimate the *Q*-matrix. Previous identifiability results are inherently disconnected from the estimation methods due to their existence proof nature: after proposing the identifiability conditions for CDMs, existing studies often proceed to estimate the *Q*-matrix and parameters using likelihood-based or Bayesian procedures. More specifically, frequentist methods often impose a sparsity-inducing penalty on continuous parameters and let the estimated sparsity pattern inform the *Q*-matrix structure (Chen et al., 2015; Xu and Shang, 2018), while Bayesian methods incorporate carefully designed MCMC sampling steps to sample only from the identifiable space of *Q*-matrices (Chen et al., 2020,1; Culpepper, 2019). Although identifiability conditions serve as a guideline for navigating *Q*-matrix estimation in these studies, the identifiability theory itself does not directly imply any specific estimation procedure.

In contrast, our constructive proof implies the possibility of developing a practical algorithm that takes the *empirical distribution tensor* 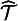 (constructed from sample proportions of observed response patterns) as input and leverages and generalizes Proposition 3 to directly estimate the *Q*-matrix. Specifically, 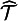 has the same size as its population counterpart 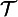 , but its entries are empirical proportions of each response pattern in the sample, instead of the population probabilities of the response patterns 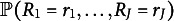 . To this end, important modifications must be made to replace the rank constraints of the unfolded population tensor with surrogate singular value conditions on the unfolded empirical tensor. Developing this procedure is out of the scope of this article, which focuses on the highly nontrivial identifiability theory as a crucial first step. We will study this direction in the future to fully realize the potential of tensor unfolding and enrich the *Q*-matrix estimation literature with efficient tensor-based methods.

## Appendix

In this Appendix, Section A provides the proofs of the main theoretical results, and Section B provides additional proofs of supporting lemmas and corollaries. Since Theorem 1 (along with Propositions 1 and 2) for the binary CDM is essentially a special case of Theorem 2 (along with Proposition 3) for the polytomous-response CDM, we focus on proving Theorem 2 and Proposition 3.

For each item  , introduce a notation  , which denotes the set of attributes required/measured by item *j*. Here, “pa” is short for “parent”, which reflects the fact that the CDM can be viewed from a directed graphical model perspective, and the set of attributes required by an item *j* can be viewed as the “parent” variables of this item that have directed edges pointing to it.

## A Proofs of the Main Results

We will need to frequently use the matrix Kronecker product “  ” and matrix Khatri-Rao product “  ” in the proofs. The Khatri-Rao product of matrices is the column-wise Kronecker product (Kolda and Bader, 2009). In particular, consider matrices  ,  ; and matrices  ,  , then  and  :

For technical convenience, we will slightly abuse notations when writing the Kronecker product and Khatri-Rao product, in that columns and rows in those products could be subject to permutations. Any of such permutations will not impact the rank properties of a matrix, so they do not affect any proof argument in this paper. Furthermore, we next state the proof of the theoretical results under the special case where all items have the same number of response categories  . We emphasize that this is only for notational simplicity, and all proof arguments in the following indeed remain exactly the same when the  ’s are allowed to differ across different *j*.

We first state three useful lemmas. Lemma A.1. Suppose  is an index set satisfying that when *j* ranges in *M*, the *M* sets  are mutually disjoint. Then, we have

(A.1)

Now consider an arbitrary set  . For any set  such that  with  , we have

(A.2)

Moreover, for any  and  and any  , we have

(A.3)

Lemma A.2. Suppose  are two disjoint sets that satisfy  ,  and  has full column rank  . Then

(A.4)

Lemma A.3. For  with  , the following holds for generic parameters:

Lemma A.4 (Lemma 3.3 in Stegeman and Sidiropoulos (2007)). Consider two matrices  of size  and  of size  .

- If  or  , then  .
- If  and  , then

### A.1 Proof of Proposition 3(a) (and Proposition 1)

The size of the unfolding  is  . If  (that is,  and  for all  ), then we can write  as

Due to the above matrix factorization of the unfolded tensor,  must hold, which proves the “if” part of Proposition 1. Next consider the “only if” part. Suppose  does not hold for any  , then  is not a singleton set, so  . In this case,

(A.5)

We next examine the rank of each of the three matrices  ,  , and  on the right-hand side of the above expression.

*First, consider*  , which is a  matrix. We apply Lemma A.3 to obtain  holds for generic parameters.

*Second, consider*  . For generic distribution of the latent variables  , the matrix  has rank  . That is,  in (A.5) generically has full rank  , which is greater than  .

*Third, consider*  . we have an important observation that when  contains two disjoint copies of  and  , the  submatrix  contains at least one submatrix of  after some row permutation. To see this, note that if  , then *either*  and  are both singleton sets with  , *or* at least one of  and  is not a singleton set. In either of these two cases, there exists a set  such that  and  . Applying Lemma A.1 gives

so  generically has full column rank  . Since  , we can write

Next, we will use Lemma A.4 to proceed with the proof. One implication of Lemma A.4(b) is that for two matrices  and  with the same number of columns, if  has full column rank and  does not contain any zero columns, then  , so  has full column rank. Note that  has full column rank and  does not contain any zero column vectors since each column of it is a conditional probability vector. So by Lemma A.4, we obtain that  has full column rank  generically. So,  in (A.5) has full row rank  generically.

Going back to (A.5), since both  and  have full rank, the rank of  equals the rank of  , which is greater than  . Now we have proved the “only if” part of the proposition that  generically if  is not a singleton set. The proof of Proposition 3(a) and Proposition 1 is complete.

### A.2 Proof of Proposition 3(b) (and Proposition 2)

Define notations  and *D*:

We first prove the “only if” part of Proposition 3(b) (and Proposition 2). It suffices to show that if  , then  . Suppose  , then  and hence  . In this case, we have the following decomposition,

Since the first matrix factor above has  columns, the above decomposition clearly shows that  .

We next prove the “if” part of Proposition 3(b) (and Proposition 2). Since  and  , we can rewrite  . So we have

(A.6)

First, the matrix factor  is a Kronecker product of  matrices, each of which has size  and has full column rank *H* for generic parameters. So,  has full rank  generically. Since  , we next will prove

(A.7)

in order to show  in Proposition 2. Denote by  the submatrix of *Q* with row indices ranging in  and column indices ranging in  . Without loss of generality, after some column permutation  can be written as follows and we denote it as  for convenience:

in other words, after some column permutation we can always write  such that its first column corresponds to the latent variable  under our current consideration, and its remaining columns correspond to all the other parent latent variables of  . Write  as

where the second matrix factor above  has size  . We next use Lemma A.3 to lower bound the rank of  . Recall that  . Define  and  , then  and  . The sets  and  satisfy that  and  is a singleton set  ; additionally,

has full column rank  . Therefore, we can use Lemma A.3 to obtain  According to the argument right after (A.7), it holds that

We now go back to the decomposition in (A.6) that  . The rank of the  diagonal matrix  is equal to  for generic parameters. The rank of the  matrix  is also equal to  for generic parameters, because  . Both  and  have full rank generically by Assumption 2. Therefore,  generically. This proves the “if” part of Proposition 3(b) (and Proposition 2). Proof of Theorems 1 and 2. It suffices to prove Theorem 2 for the polytomous CDMs because the binary CDMs in Theorem 1 are special cases of the former. Proposition 3 has the following implications. If one exhaustively investigate all pairs of items indexed by  , then we can exactly obtain *K* disjoint sets of item indices  with the following property: For any  , it holds that

The above argument can also uniquely identify the maximum number of latent variables *K*. This is because *K* is equal to the number of all the nonoverlapping sets  such that for any  for each *k*, it holds that  . After exhaustively inspecting  for all pairs of item indices  , the number of such nonoverlapping sets is uniquely determined and *K* is uniquely determined constructively. Thus far, we have identified all the row vectors indexed by  . This is exactly the set of all single-attribute items among the *J* items. Now, using the assumption that the true *Q*-matrix contains at least two single-attribute items for each attribute, we can permute the already identified row vectors in *Q* so that it can be written as  . Further, for any item *j* that is not a single-attribute item, in order to determine whether  for each  , we consider the following marginalized unfolding

Here both *B* and *D* are non-overlapping sets with cardinality *K*, so  is a  matrix. Since  , part (b) of Proposition 1 implies that  holds if and only if  . Therefore, examining whether the rank of  exceeds  exhaustively for all non-single-attribute items *j* and all  will identify those row vectors of the *Q*-matrix corresponding to these items. Now, we have identified all quantities, including *K* and the *Q*-matrix, and the proof is complete.

## B Proof of Supporting Results

### B.1 Proof of Lemma A.1

We first prove (A.1). By the conditional independence of the observed variables given the latent parents, we have

which implies for any observed pattern  and any latent pattern  , it holds that

Since  for  are disjoint singleton sets, by following the definition of the Kronecker product of matrices, the above factorization implies  and proves (A.1).

We next prove (A.2). First, note that  has size  , while  has size  and  has size  . So, the sizes of matrices on the left-hand side and the right-hand side of (A.2) match each other. Further, since  , we know that the conditional distribution of  given  only depends on those latent variables belonging to the index set  ; in other words,

where  and  are a subvectors of  . The above fact implies that, although  has  columns indexed by all possible configurations of  , actually  contains only  unique columns. Each unique column is indexed by a unique pattern the subvector  takes in  . So, the matrix  horizontally stacks  repetitive blocks, each of which equals  . So, we have

We next prove (A.3). Since  holds,  and  are conditionally independent given  . So, we can use Lemma 12 in Allman et al. (2009) to direct obtain the conclusion that  . The proof of Lemma A.1 is complete.

### B.2 Proof of Lemma A.2

Since  , we use Lemma A.1 to write

and

(B.1)

Define notation

For each fixed pattern  , the  is a  matrix with rows indexed by all possible configurations of  and columns by all possible configurations of  . By the condition in the lemma, matrix  has full column rank  . For notational simplicity, denote this number  . After possibly rearranging the columns of  and  via a common permutation, we can write

Therefore, the matrix  in (B.1) can be written as

(B.2)

Next, we will prove that the matrix  has rank at least  under Assumption 2. Denote the columns of  by  , and the columns of  by  . Note that by definition, each  is a vector describing the conditional distribution of  given a specific configuration of  when  is fixed to be  . Since  , there exists some  but  . Under the non-DINA/DINO assumption in Definition 3, there exist  different vectors  where  differ only in the entry corresponding to  , in which there exist  and some  such that

(B.3)

Suppose that there exists a vector  such that

Rearranging the terms above gives

(B.4)

Define a matrix  whose *r*-th column vector is  , and for any  and  , the *m*-th column vector of  is  . Define a *R*-dimensional vector  with  and  for any  and  . Then (B.4) can be rewritten as

(B.5)

Since  above is not a zero vector, we have that  . We next will show that this necessarily implies  .

We introduce the definition of the Kruskal rank. The Kruskal rank of a matrix is the largest integer *M* such that every *M* columns of this matrix are linearly independent. Denote the Kruskal rank of any matrix  by  . It is easy to see that if a matrix has full column rank, then its Kruskal rank equals its rank. Also, a matrix  contains a zero column vector if and only if  .

Now consider the matrix  in (B.5). Since  has full column rank *R*, then  . For any  , the *m*-th column vector of  is a conditional probability vector for  given a fixed pattern of  , therefore  . So, if  , then  does not have any zero column vectors and  . In this case, the following holds by Lemma A.4(b),

Then,  , which contradicts with (B.5) because the vector  there is not a zero vector with  and hence  should not have full column rank. This contradiction implies that

Now note that each of  and  is a conditional probability vector describing the distribution of  given a fixed pattern of  . Therefore,  , and we further have  . Plugging the above expression  back into (B.4) further gives

(B.6)

We next use Lemma A.4 to show that  are  linearly independent vectors. To show this, first note that (B.6) can be written as

where matrix  contains all but the *r*-th column of matrix  , matrix  contains all but the *r*-th column of matrix  , and vector  contains all but the *r*-th entry of the vector  . Now, because  and  , Lemma A.4(b) implies that

Since  contains  columns, the above inequality gives that  . In other words, the  vectors in  are linearly independent. Therefore, from (B.6) we have that  for all  . Recall we aim to examine whether there exists a nonzero vector  such that (B.4) holds, and we have already shown that under (B.4), the only two potentially nonzero entries in the vector  are  and  . If  , then  , and it indicates

The above equality contradicts the earlier (B.3) that  which follows from Assumption 2, therefore  must hold. In summary, we have shown that the  vectors  are linearly independent, which means the matrix  in (B.2) contains at least  linearly independent column vectors and  . The proof of Lemma A.2 is complete.

### B.3 Proof of Lemma A.3

Consider all possible cases: Case (a),  ; Case (b),  with  ; Case (c),  with  ; and Case (d),  and  with  ,  . Denote  .

**Case (a):** Since  , we have

so  .

**Case (b):** We have  , so both  and  are generic conditional probability matrices without particular structures (i.e., without equality between certain columns). We can write

Under the non-DINA/DINO assumption in Definition 3, we apply Lemma 13 in Allman et al. (2009) to obtain that  holds for generic parameters.

**Case (c):**  with  . Denote  . We can apply Lemma A.1 to write

(B.7)

where each  denotes a  submatrix of  . Here,  indexes a specific configuration of  , and the  columns of  are indexed by all possible configurations  can take. Therefore, each matrix  collections the conditional probability vectors of  when fixing  and varying  . Note that  is a generic matrix without equality constraints between certain columns. Therefore, we apply Lemma 13 in Allman et al. (2009) to obtain that the following holds for generic parameters:

Now, if  , then the above inequality gives  , which further implies  . So, it remains to consider the case with  . In this case,  has full column rank  by Assumption 2. Define  and  , then the conditions  and  in Lemma A.2 are satisfied. So Lemma A.2 gives that the rank of  is greater than  . In summary, in Case (c),  .

**Case (d):**  and  with  ,  . Note that  and  imply that both  and  contain at least two elements. First consider the  matrix

which takes the same form as (B.7) in case (c) and falls into the same setting considered in case (c). Therefore, we know that  holds generically. Now, we claim that this matrix  has the following linear transformation relationship with the original matrix  :

(B.8)

where  has mutually orthogonal columns and hence full column rank. To see that this claim is true, we only need to note that for any vectors  and  , it holds that

This equality means that to get the smaller conditional probability table  , we can just sum up appropriate columns in the larger conditional probability table  , where the  matrix  has binary entries that reflect this summation. Specifically, for any  and  , the corresponding entry in *S* is

Since each column in  is indexed by a different configuration  , the column vectors of  have mutually disjoint support and are orthogonal. Now we have shown that the claim about (B.8) is correct. Therefore, (B.8) implies that

Now we have proved the conclusion of Lemma A.3 for all possible cases (a), (b), (c), and (d). The proof is complete.

## References

[r1] Allman, E. S. , Matias, C. , and Rhodes, J. A. (2009). Identifiability of parameters in latent structure models with many observed variables. The Annals of Statistics, 37(6A):3099–3132.

[r2] Chen, J. and de la Torre, J. (2018). Introducing the general polytomous diagnosis modeling framework. Frontiers in Psychology, page 1474. 10.3389/fpsyg.2018.01474 PMC6113892 30186195

[r3] Chen, Y. , Culpepper, S. , and Liang, F. (2020). A sparse latent class model for cognitive diagnosis. Psychometrika, 85(1):1–33. 10.1007/s11336-019-09693-2 31927684

[r4] Chen, Y. , Culpepper, S. A. , Chen, Y. , and Douglas, J. (2018). Bayesian estimation of the DINA Q matrix. Psychometrika, 83(1):89–108. 28861685 10.1007/s11336-017-9579-4

[r5] Chen, Y. , Liu, J. , Xu, G. , and Ying, Z. (2015). Statistical analysis of Q-matrix based diagnostic classification models. Journal of the American Statistical Association, 110(510):850–866. 26294801 10.1080/01621459.2014.934827 PMC4539161

[r6] Culpepper, S. A. (2019). An exploratory diagnostic model for ordinal responses with binary attributes: identifiability and estimation. Psychometrika, 84(4):921–940. 31432312 10.1007/s11336-019-09683-4

[r7] Culpepper, S. A. (2023). A note on weaker conditions for identifying restricted latent class models for binary responses. Psychometrika, 88(1):158–174. 35896935 10.1007/s11336-022-09875-5

[r8] de la Torre, J. (2011). The generalized DINA model framework. Psychometrika, 76:179–199.

[r9] DiBello, L. V. , Stout, W. F. , and Roussos, L. A. (2012). Unified cognitive/psychometric diagnostic assessment likelihood-based classification techniques. In Cognitively diagnostic assessment, pages 361–389. Routledge.

[r10] Fang, G. , Liu, J. , and Ying, Z. (2019). On the identifiability of diagnostic classification models. Psychometrika, 84(1):19–40. 30673967 10.1007/s11336-018-09658-x

[r11] Gu, Y. (2023). Generic identifiability of the DINA model and blessing of latent dependence. Psychometrika, 88(1):117–131. 36167947 10.1007/s11336-022-09886-2

[r12] Gu, Y. and Xu, G. (2020). Partial identifiability of restricted latent class models. Annals of Statistics, 48(4):2082–2107.

[r13] Gu, Y. and Xu, G. (2021). Sufficient and necessary conditions for the identifiability of the 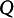 -matrix. Statistica Sinica, 31:449–472.

[r14] Henson, R. A. , Templin, J. L. , and Willse, J. T. (2009). Defining a family of cognitive diagnosis models using log-linear models with latent variables. Psychometrika, 74:191–210.

[r15] Junker, B. W. and Sijtsma, K. (2001). Cognitive assessment models with few assumptions, and connections with nonparametric item response theory. Applied Psychological Measurement, 25:258–272.

[r16] Kolda, T. G. and Bader, B. W. (2009). Tensor decompositions and applications. SIAM Review, 51(3):455–500.

[r17] Kruskal, J. B. (1976). More factors than subjects, tests and treatments: an indeterminacy theorem for canonical decomposition and individual differences scaling. Psychometrika, 41:281–293.

[r18] Kruskal, J. B. (1977). Three-way arrays: rank and uniqueness of trilinear decompositions, with application to arithmetic complexity and statistics. Linear Algebra and its Applications, 18(2):95–138.

[r19] Liu, J. , Xu, G. , and Ying, Z. (2013). Theory of the self-learning Q-matrix. Bernoulli: official journal of the Bernoulli Society for Mathematical Statistics and Probability, 19(5A):1790–1817. 24812537 10.3150/12-BEJ430 PMC4011940

[r20] Liu, Y. and Culpepper, S. A. (2024). Restricted latent class models for nominal response data: Identifiability and estimation. Psychometrika, 89(2):592–625. 38114767 10.1007/s11336-023-09940-7

[r21] Ma, W. and de la Torre, J. (2016). A sequential cognitive diagnosis model for polytomous responses. British Journal of Mathematical and Statistical Psychology, 69(3):253–275. 27317397 10.1111/bmsp.12070

[r22] Maris, E. (1999). Estimating multiple classification latent class models. Psychometrika, 64:187–212.

[r23] Rupp, A. A. and Templin, J. L. (2008). Unique characteristics of diagnostic classification models: A comprehensive review of the current state-of-the-art. Measurement, 6(4):219–262.

[r24] Stegeman, A. and Sidiropoulos, N. D. (2007). On Kruskal’s uniqueness condition for the Candecomp/Parafac decomposition. Linear Algebra and its Applications, 420:540–552.

[r25] Tatsuoka, K. K. (1983). Rule space: an approach for dealing with misconceptions based on item response theory. Journal of Educational Measurement, 20:345–354.

[r26] Templin, J. L. and Henson, R. A. (2006). Measurement of psychological disorders using cognitive diagnosis models. Psychological Methods, 11(3):287. 16953706 10.1037/1082-989X.11.3.287

[r27] von Davier, M. (2008). A general diagnostic model applied to language testing data. British Journal of Mathematical and Statistical Psychology, 61:287–307. 17535481 10.1348/000711007X193957

[r28] von Davier, M. (2014). The log-linear cognitive diagnostic model (LCDM) as a special case of the general diagnostic model (GDM). ETS Research Report Series, 2014(2):1–13.

[r29] von Davier, M. and Lee, Y.-S. (2019). Handbook of diagnostic classification models. Cham *:* Springer International Publishing.

[r30] Wayman, E. A. , Culpepper, S. A. , Douglas, J. , and Bowers, J. (2025). A restricted latent class model with polytomous attributes and respondent-level covariates. Behaviormetrika, pages 1–29.

[r31] Xu, G. (2017). Identifiability of restricted latent class models with binary responses. The Annals of Statistics, 45(2):675–707.

[r32] Xu, G. and Shang, Z. (2018). Identifying latent structures in restricted latent class models. Journal of the American Statistical Association, 113(523):1284–1295.

[r33] Xu, G. and Zhang, S. (2016). Identifiability of diagnostic classification models. Psychometrika, 81(3):625–649. 26155755 10.1007/s11336-015-9471-z

